# An Inflammatory Story: Antibodies in Tuberculosis Comorbidities

**DOI:** 10.3389/fimmu.2019.02846

**Published:** 2019-12-09

**Authors:** Milla R. McLean, Lenette L. Lu, Stephen J. Kent, Amy W. Chung

**Affiliations:** ^1^Department of Microbiology and Immunology, Peter Doherty Institute for Infection and Immunity, University of Melbourne, Melbourne, VIC, Australia; ^2^Division of Infectious Disease and Geographic Medicine, Department of Internal Medicine, University of Texas Southwestern Medical Center, Dallas, TX, United States; ^3^Infectious Diseases Department, Melbourne Sexual Health Centre, Alfred Health, Central Clinical School, Monash University, Brisbane, VIC, Australia; ^4^ARC Centre of Excellence in Convergent Bio-Nano Science and Technology, University of Melbourne, Melbourne, SA, Australia

**Keywords:** antibody, glycosylation, tuberculosis, HIV, diabetes, kidney disease, co-infection, inflammation

## Abstract

*Mycobacterium tuberculosis* (*Mtb*) resides in a quarter of the world's population and is the causative agent for tuberculosis (TB), the most common infectious reason of death in humans today. Although cellular immunity has been firmly established in the control of *Mtb*, there is growing evidence that antibodies may also modulate the infection. More specifically, certain antibody features are associated with inflammation and are divergent in different states of human infection and disease. Importantly, TB impacts not just the healthy but also those with chronic conditions. While HIV represents the quintessential comorbid condition for TB, recent epidemiological evidence shows that additional chronic conditions such as diabetes and kidney disease are rising. In fact, the prevalence of diabetes as a comorbid TB condition is now higher than that of HIV. These chronic diseases are themselves independently associated with pro-inflammatory immune states that encompass antibody profiles. This review discusses isotypes, subclasses, post-translational modifications and Fc-mediated functions of antibodies in TB infection and in the comorbid chronic conditions of HIV, diabetes, and kidney diseases. We propose that inflammatory antibody profiles, which are a marker of active TB, may be an important biomarker for detection of TB disease progression within comorbid individuals. We highlight the need for future studies to determine which inflammatory antibody profiles are the consequences of comorbidities and which may potentially contribute to TB reactivation.

## Introduction

*Mycobacterium tuberculosis (Mtb)* is a leading cause of mortality and morbidity across the globe with an estimated 10 million new infections annually and a quarter of the world's population infected (1). The spectrum of tuberculosis (TB) in humans is most widely characterized by two clinical states: active TB and latent TB. Individuals with active TB exhibit symptoms such as hemoptysis, fever and weight loss with detectable bacteria, while individuals with latent TB are not overtly clinically ill, have no detectable *Mtb*, and therefore no transmission risk (see Table 1). An important reason for the significant burden of TB today, is our poor understanding of the human immune response to *Mtb* infection. More specifically, why 5–10% of infected individuals progress to active disease while others remain latent is not known (4). In many low to middle income countries where TB is endemic, there exists a double burden of such communicable diseases with the rapid rise in chronic and non-communicable diseases (5). The HIV-1 and TB co-infection syndemic is highly alarming (6) with TB being the leading cause of death in people living with HIV-1 (7). An estimated 49% of HIV-1 infected individuals are unaware of their co-infection and post-mortems on HIV-1 infected adults showed 64% had evidence of disseminated *Mtb* (7, 8).

**Table 1:** Clinical spectrum of TB (2, 3).

**Active TB**	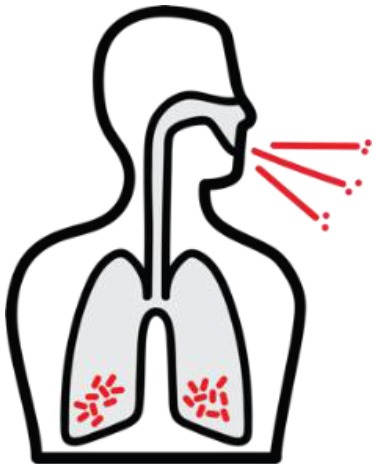	• Severe symptoms • High transmission • Smear and culture *Mtb* positive • Highest bacillary burden **Diagnostically** 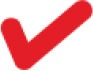 TST positive 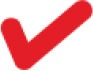 IGRA positive (if immunocompetent) 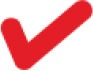 Chest X-Ray positive
**Subclinical TB**	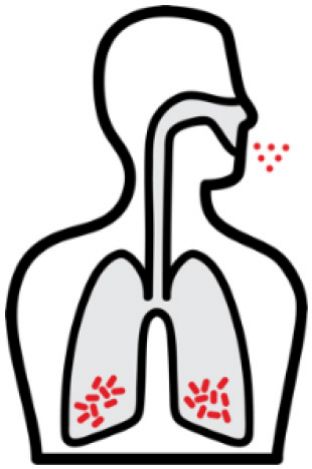	• Mild symptoms or asymptomatic • Intermittent transmission • Smear or culture *Mtb* positive • Moderate bacillary burden **Diagnostically** 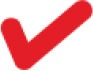 TST positive 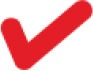 IGRA positive (if immunocompetent) 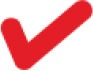 Chest X-Ray positive
**Incipient TB**	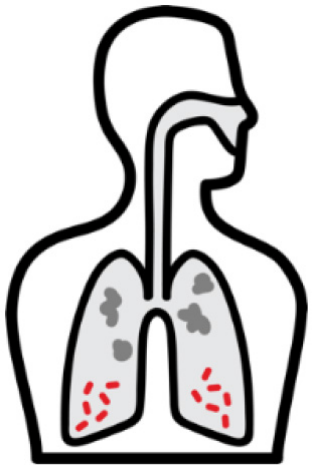	• Asymptomatic • Low transmission • Culture negative • Moderate bacillary burden **Diagnostically** 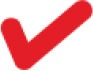 TST positive 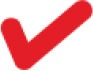 IGRA positive (if immunocompetent) 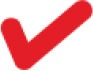 Chest X-Ray showing upper-lobe opacities
**Latent TB**	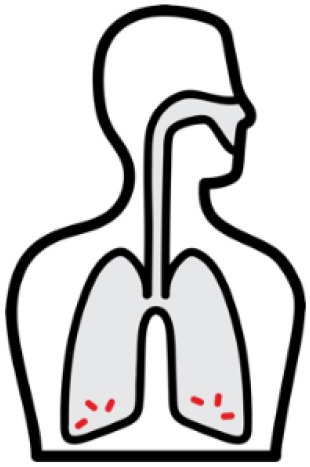	• Asymptomatic • Low transmission • Smear negative and culture negative • Low bacillary burden **Diagnostically** 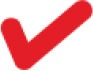 TST positive 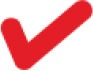 IGRA positive (if immunocompetent) 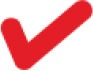 Chest X-Ray negative
**“Resisters”**	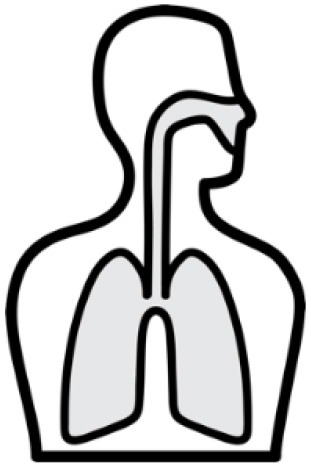	• Asymptomatic • No transmission • Smear and culture negative • Low bacillary burden **Diagnostically** 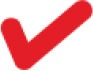 TST Negative 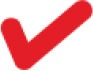 IGRA Negative (if immunocompetent) 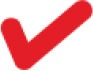 Chest x-Ray Negative

*TST, Tuberculin Skin Test; IGRA, interferon-γ-release assay*.

Studies in the context of comorbid conditions in humans as well as animal models have helped identify some of the mechanisms underlying this transition from latent infection to active disease (9). Specifically, the HIV epidemic has highlighted T cell function as an important component of immune protection. Besides HIV, there remains many other significant conditions in which the risk of *Mtb* acquisition and or progression from latent infection to active disease is increased (10–12).

Diabetes as an epidemiological risk factor for TB is well-reported (13). Spanning back to 1947, a review of diabetes and *Mtb* co-infection reported that 50% of diabetics succumbed to pulmonary TB (10). In recent years the epidemic has grown, with the number of individuals with Diabetes-TB overtaking those with HIV-TB (14); which can be attributed in part, to the positive impacts that antiretroviral therapy is having on reducing TB-HIV co-infection (15). Moreover, a recent multi-country cohort study found that patients with Diabetes-TB had more severe TB disease compared to individuals without diabetes (16). Thus, diabetes presents an independent risk factor for acquisition of *Mtb* infection and also progression of disease.

Chronic Kidney Disease (CKD) associated with and also independent of diabetes, represents an additional risk factor for TB. Patients with late-stage CKD, called end stage renal disease (ESRD) requiring dialysis have a ~50-fold higher risk of latent TB reactivation (11). Additionally, TB contributes to mortality in individuals with CKD, and with a global rise in total CKD [18.4% increase since 2005 (17)], it is expected that cases of CKD/TB comorbidity will increase in prevalence (18, 19). The immunological causes of TB reactivation, however, are not well-understood in patients with these chronic diseases and thus we have a significant gap in our understanding of the immune response to *Mtb* infection.

While the importance of T cells in TB control is firmly established, the fact that other comorbid conditions and healthy individuals with intact T cell responses (as far as we know) can progress from latent infection to active TB, suggests that there are additional immune mediated mechanisms of protection. Moreover, T cell based diagnostics fail to distinguish between latent and active TB and these tests cannot reliably detect TB in HIV-1 infected individuals (20, 21). Finally, the BCG vaccine inducing potent T cell responses is sub optimally protective (22, 23). Thus, a broader understanding of the immune response to TB is needed.

**Figure 1:**
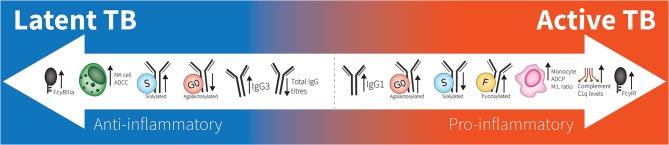
Spectrum of antibodies in latent to active TB. Latent responses are in comparison to active TB. FcγRIIIa increases are due to affinity binding while FcγRI increases are due to elevated expression. “M:L ratio” is Monocyte:Lymphocyte ratio. No differences are seen between healthy individuals and latent TB.

In recent years, there has been more focus on investigating the role of antibodies and innate cells in TB infection and disease (24, 25). This interest in humoral immunity in *Mtb* is evidenced by a mounting number of studies that have identified specific antibody targets, and structural or functional differences that are observed during different TB disease states (26–30). For example, while *Mtb*-specific IgG titers alone have been insufficient in distinguishing between latent and active TB, refinement of these correlates using additional isotypes, subclasses, and other Fc modifications are underway (see Figure 1) (31–35). Moreover, incorporating data including multiple instead of single *Mtb* antigens as well as Fab affinity and avidity to *Mtb* targets may improve sensitivity and specificity (36–40). Thus, assessing more specific antibody features may improve our understanding of humoral immune correlates of infection and disease.

**Figure 2:**
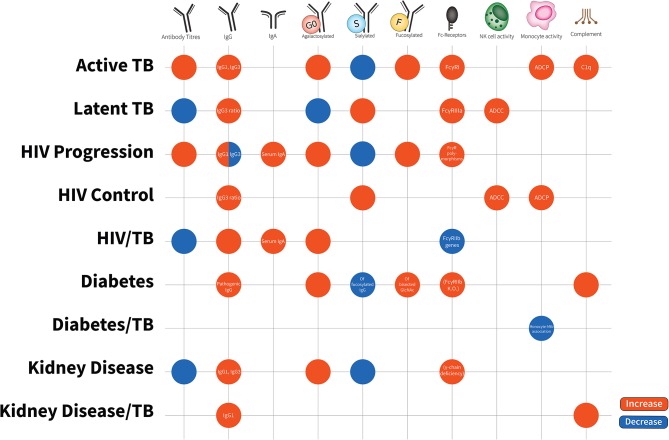
Antibody characteristics (isotypes, subclasses, glycosylation patterns, cellular activity, and Fc-receptor patterns) in latent TB, active TB, HIV disease progression, HIV disease control, HIV/TB co-infection, Diabetes, Diabetes/TB comorbidity, Kidney Disease, Kidney Disease/TB comorbidity. Increases are in red; decreases are in blue. Latent TB is indifferent to uninfected individuals and comparisons are to active TB. HIV progression is compared to viremic controllers. HIV/TB is compared to single infection alone. Diabetes/TB is compared to diabetes alone. Kidney disease/TB is compared to diabetes alone. FcγRIIIa increases are due to affinity binding while FcγRI increases are due to elevated expression. Blank spots are due to no changes or no evidence in this area.

This review examines the antibody profiles (isotypes, subclasses, functions, and post translational modifications) in TB and diseases in which TB has high rates of reactivation, focusing primarily on HIV-1, type 2 diabetes mellitus (T2DM), and CKD (see Figure 2). We draw together what is known about antibodies and their role in inflammation in infectious and non-communicable diseases, a novel take on examining humoral immunity in co-infection. Further, we discuss antibody characteristics described in the limited studies of *Mtb* comorbidity cohorts. Understanding antibody characteristics in *Mtb* infection, conditions where TB reactivates and their comorbidities will assist in drawing links between immune states in each disease and potential common mechanisms of TB reactivation (25). We speculate whether these antibody characteristics may ultimately find utility as biomarkers in assessing a patient's real-time risk of TB reactivation to personalize treatment plans and follow-up.

## Antibody Structure

The antibody glycoprotein exists within the immunoglobulin (Ig) superfamily. The Y-shaped heterodimeric protein consists of two light chains (C_L_) and two heavy chains (C_H_). Its tertiary structure can be functionally divided into the Fab region proximally and the Fc region distally, between which lies a flexible hinge region (41). Antibodies can be viewed as a “bridge” between innate and adaptive immunity since they have the capacity to bind targets via the Fab region and activate innate immune effector cells via Fc-receptors (42, 43). The functional capacity of an antibody is determined by a range of characteristics; including the antibody isotype and subclass, but also the many complex modifications that ensue post-translationally.

### Antibody Isotypes and Subclasses

Within the range of human antibodies, there exists 5 isotypes; IgM, IgD, IgG, IgE, and IgA. IgG predominates in human serum and can be subclassed into IgG1, IgG2, IgG3, and IgG4 in order of most to least abundance, while IgA predominates in mucosal sites and can be subclassed into IgA1 and IgA2 (44, 45). IgG, IgA, and IgE isotypes can be further classified into allotypes which exist as genetic polymorphisms within the C_L_ and C_H_ (46, 47).

Antibody isotypes and subclasses differentially interact with Fc receptors (FcR) thus determining the range of effectors cells activated and their immune response. Antibody isotype and subclass class switch recombination is modulated by B cell factors and toll-like receptor stimuli [e.g., Activation-induced deaminase and CPG (48)] and is often influenced by T cell help (e.g., CD40L) and specific cytokines secreted by a range of classical T helper cells and non-classical T cells such as NK T and gamma-delta T cells (49–51). In general, extracellular bacterial infections skew subclass responses toward IgG1 and IgG2, which are elicited in response to the high polysaccharide and protein composition of these bacteria. These antibodies have the ability to neutralize bacterial toxins, opsonize capsular polysaccharides for phagocytosis, as well as activate the complement membrane-attack complex for bacterial lysis (52–56). Viral infections and intracellular bacteria primarily elicit IgG1 and IgG3 subclasses. Antiviral IgG1 and IgG3 antibodies are commonly divided into neutralizing antibodies which bind free virus preventing cell entry and non-neutralizing antibodies, though there is a growing awareness of neutralizing antibodies with the capacity to activate non-neutralizing functions (57, 58).

### Antibody Glycosylation

Post-translational modifications of antibodies have substantial downstream effects on their engagement of cells and cellular diversity, antibody localization, protein half-life, and stability (59, 60). The most comprehensively studied in relation to antibodies is N-glycosylation, where research mainly focuses upon how glycosylation of antibodies can improve monoclonal antibody therapeutics (61, 62).

Glycosylation is the covalent addition of sugar moieties to portions of an antibody in the endoplasmic reticulum and golgi. The glycan molecules which can be added include galactose, sialic acid, fucose, and bisecting N-acetylglucosamine (GlcNAc) (60). There is a key N-glycosylation site on all IgG Fc regions at an Asn-297 residue of IgG CH_2_ heavy chains. In addition, approximately 10–20% of serum antibody Fab regions contain N-linked glycans, while O-linked glycans are also present in the hinge region of IgG3 and IgA1 (63, 64).

Glycosylation of IgG holds the two heavy chains in an open confirmation, allowing binding to Fcγ-receptors (65). Fully deglycosylated IgG have reduced capacity to engage most Fc Receptors (66–68). Agalactosylated antibodies (G0) are upregulated in inflammatory diseases that are commonly associated with enhanced presence of pro-inflammatory cytokines in serum. These agalactosylated G0 antibodies have the capacity to activate alternative and mannose-binding lectin complement pathways (69). Bisecting GlcNAc on IgG1 has been associated with increased FcγRIIIa binding (70) while the presence of core fucose residues reduces FcγRIIIa binding and subsequent ADCC (71). On the other hand, sialylated antibodies enhance anti-inflammatory activities via engagement with DC-Sign and inhibitory FcγRIIb binding (64, 72). Recent studies suggest that antibody glycosylation can also affect antigen binding affinity and affinity maturation (73, 74).

### Antibody Fc Functions

Antibody Fc functions link the adaptive and innate immunity. Fc-functional antibodies can simultaneously bind a target via the Fab region and an innate immune FcR effector cell via the Fc region. Cross-linking of these antibodies on the surface of effector cells leads to a downstream activating signaling cascade, inducing a range of effector functions including targeted cell killing, otherwise known as antibody-dependent-cellular-cytotoxicity (ADCC), antibody-dependent-phagocytosis (ADP), cytokine, chemokine and/or enzyme release (75, 76). The FcR syntax for IgA is FcαR, IgE is FcεR, and IgG is FcγR, of which there exists different isoforms (42). Different effector cells can express a different combination of activating FcγR (e.g., FcγRI/IIa/IIc/IIIa/IIIb) and/or inhibitory FcγR (FcγRIIb) receptors on their surface. Further, IgG subclasses differentiate in their binding affinity to different FcγRs with IgG3 and IgG1 being subclasses with the highest affinity (77, 78). The affinity, diversity, and polymorphisms of FcγR and IgG subclasses is reviewed elsewhere (79).

## Tuberculosis (TB)

*Mtb* infection in humans produces a spectrum of clinical and subclinical disease. This continuum can be loosely grouped into active TB, subclinical TB, incipient TB, latent TB, and “Resisters.” Individuals with active TB have detectable bacillary burden by culture or PCR and commonly have a positive interferon-γ (IFN-γ) releasing assay (IGRA) or tuberculin skin test (TST) with cough, weight loss, and fever. Subclinical TB is defined as asymptomatic disease but with a loss of bacterial containment which can be observed with inflammation as detected radiographically by positron emission tomography (PET). At the other end of the spectrum, latent TB is identified as persistent asymptomatic infection, TST or IGRA positive with no transmission capacity (2). Unlike subclinical TB, individuals with latent infection do not demonstrate areas of PET avidity consistent with active disease. Finally, a subset of TST and IGRA negative individuals have been identified called “Resisters” who appear to have non canonical immune responses to *Mtb* antigens in the context of high levels of exposure (80). Clinical and diagnostic details of these TB disease groups have been recently reviewed by Furin et al. (3) and are summarized in Table 1.

### Pro-Inflammatory Indicators in *Mycobacterium tuberculosis* Infection

While delineating mechanisms of protection is important in understanding TB pathogenesis, enhancement of disease is a physiologically important component of the host response. The full spectrum of clinical presentations from latency to disseminated extra-pulmonary TB initiates varying and complex host-pathogen interactions (81, 82). The role of IFN-γ releasing CD4+ T cells is complex and somewhat paradoxical. IFN-γ knock-out mice succumb to early *Mtb* infection compared to wildtype (83, 84). In humans, IFN-γ releasing T_H_1 CD4+ cells are widely recognized as the predominate cell-type that responds to *Mtb* infection with their numbers increasing post-infection and at granuloma sites, where bacteria are surrounded by innate and adaptive immune cells (85, 86). Mutations in IFN signaling are associated with non-tuberculous mycobacterial infections (87), and the use of inhibitors of TNF-α, another important inflammatory cytokine, as disease modifying antirheumatic drugs in patients with autoimmune diseases is associated with increased risk of TB (88, 89). The induction of IFN-γ releasing cells has also been a primary aim of many TB vaccine efforts with vaccine correlate studies focusing on eliciting high T cell release of type 1 cytokines including IFN-γ, TNF-α, and IL-2 (90–93). Moreover, a recent study into BCG vaccine-induced IFN-γ releasing T cells reported that these cells protect if expanded in response to vaccination (94). Thus, multiple lines of evidence in animal models and humans across *Mtb* and BCG demonstrate the protective potential of IFN-γ and TNF-α.

However, more recent evidence brings to light that IFN-γ, and also TNF-α, in TB is not solely protective and the landscape is in fact more complex. A growing number of studies correlate IFN-γ releasing T_H_1 cells (22, 23, 93, 95, 96) and TNF (97) post-infection with enhanced disease burden and reduced survival. Indeed, with PD-1 inhibitor blockade in pre-clinical models and in humans, *Mtb* specific CD4+ T cells producing IFN-γ increase in the blood, correlating with progression of TB disease (98, 99). Finally, an immune correlates study looking at BCG vaccination in infants, showed that the induction of type-1 cytokines released from peripheral blood CD4+ T cells co-expressing IFN-γ, TNF-α, and IL-2 did not correlate with TB protection (100). This lack of correlation is also seen in tissue specific *Mtb-*reactive CD4+ T cell responses in mice (22). Extending from children to adults, double positive TNF-α+/IFN-γ+ CD4+ T cells and single positive TNF-α+ CD4+ T cells were substantially elevated in active compared to latent TB (101, 102). One possible explanation for this complexity is that the timing of *Mtb*-specific CD4+ T cell responses may be critical to their role in TB protection or burden as in whether the cell population and subsequent inflammation expands before or after *Mtb* infection (25, 103). Thus, pre-existing T cell responses induced by vaccination and or exposure may protect against disease, but expansion of T cells in the context of disease progression may not be helpful. In fact, phagocytosis alone stimulates substantial IL-12 release which alongside IFN-γ drives a switch to the inflammatory T_H_1 phenotype (104). As such, pro-inflammatory cytokine profiles, including IFN-γ, IL-1, IL-2, IL-13, and TNF-α, mark the state of active TB in patients (105, 106). Moreover, excess proinflammatory signaling can result in tissue remodeling and destruction (107, 108), and high levels of TNF-α can be detrimental, in some circumstances contributing to cachexia and weight loss (97, 101, 102, 109–111). However, orthogonal pro-inflammatory cytokines can be potentially protective in individuals with latent infection or those who have been highly exposed to *Mtb* (30, 112). One study looking at healthcare workers in frequent contact with TB patients, found no differences in IFN-γ release from PBMCs in response to purified protein derivative compared to culture-positive TB patients (113). In the study by Lu et al. latent TB individuals had greater IFN-γ release from *Mtb*-specific CD4+ T cells than “resisters” who reported almost no IFN-γ response despite the “resister” population having *Mtb*-specific IgM and IgG (30). The magnitude of the IFN-γ response in the resister population may be a function of lower antigen load, as “resisters” are thought to have no replicative bacteria. How antibodies may inform on these inflammatory states remains to be fully elucidated.

### Antibody Isotypes and Subclasses in *Mycobacterium tuberculosis* Infection

In the broadest of strokes, antibody titers increase as *Mtb* burden increases, presumably due to increased antigen availability (26, 37, 114, 115). More specifically, some but not all studies show that active pulmonary TB elicits an *Mtb*-specific IgG isotype response with the elevation of both *Mtb-*specific and total IgG1 and IgG3 subclasses in serum (37, 116–118). *In vitro*, IgG1 mediates TNF-α release from human monocytes but does not increase the anti-inflammatory cytokine IL-10 (109, 110). IgG1 and IgG3 subclasses are also the main antibodies capable of activating complement (119). Consistent with these associations, complement C1q levels are higher in active TB compared to latent TB, and complement cascade molecules are upregulated 18 months before progression from *Mtb* infection to TB disease (120–122). Moreover, some monoclonal *Mtb* IgG1 can enhance bacterial replication *in vitro*, demonstrating the potential to exacerbate disease (27). Finally, polyclonal serum from *Mtb* infected rabbits can enhance BCG infection in mice (123). Thus, specific subclasses such as IgG1 and IgG3 in the context of monoclonality or polyclonality may participate in the induction of pro-inflammatory downstream consequences as seen in active TB disease and potentially favor the bacteria.

However, not all IgG is pathogenic. Passive transfer into mice of purified polyclonal IgG from some healthcare workers highly exposed to *Mtb* with detectable *Mtb* specific IgG can decrease *Mtb* burden in the lung (28). Similarly, serum from mice immunized by a variety of antigens thought to be cross reactive to *Mtb* capsular macromolecules can delay *Mtb* outgrowth from a variety of organs and increase survival time in mice (124, 125). This protective impact, along with decreased associated pulmonary pathology, can even be seen with intact intravenous immunoglobulin (IVIG) preparations of pooled polyclonal IgG from multiple healthy humans used to clinically treat autoimmune diseases (126, 127). Notably, the protective effect appears to be abrogated with enzymatic removal of glycan residues (127). Therefore, *Mtb* reactive IgG with intact glycosylation in these complex polyclonal responses may participate in controlling bacterial burden. Further studies capturing the antigen specificities and glycan signatures of these antibodies will illuminate the mechanistic basis of these findings.

Beyond IgG, IgA has been a focus of interest due to the importance of this isotype in mucosal immunity within the pulmonary compartment, the main route of TB infection, acquisition and subsequent transmission. While analysis of bronchoalveolar lavage (BAL) (128, 129) from individuals diagnosed with pulmonary TB demonstrates the presence of *Mtb*-specific IgA in addition to IgG, not all IgA is protective. In mice, transfer of B cells induced by an *Mtb* subunit mucosal vaccine that produces detectable antigen-specific IgA in the lung is not protective compared to the T cell counterparts (130). This may be in part due to the lack of the Fcα-receptor/CD89 human homolog in mice as one of the many challenges in translating between species (131). Indeed, the protective effect of intranasal delivery of one monoclonal *Mtb* IgA is dependent on the presence of CD89 expressed transgenically (132). Yet, mice deficient in IgA have increased susceptibility to *Mycobacterium bovis* in the context of subunit vaccination (133), suggesting that non FcαR mediated qualities could also be involved. In fact, intratracheal delivery of a different monoclonal *Mtb* IgA into mice has shown some protection with decreased bacterial burden and pulmonary pathology in absence of CD89 (134). Consistent with these findings, a recent *in vitro* study observed that monoclonal IgA were able to inhibit *Mtb* growth, while IgG antibodies to the same targets promoted infection (27). Intriguingly, this isotype mediated control of *Mtb* appears to be modulated via FcαR/FcγR independent mechanisms, as these results were observed when using epithelial cell lines that lack these FcR receptors. The authors hypothesize that the IgG *Mtb* uptake and infection may instead have been mediated by FcRn binding, though no mechanistic studies were conducted to confirm this. While monoclonal antibodies differ compared to polyclonal humoral immune responses in infection due to both antigen specificity and antibody glycosylation, these monoclonal studies suggest that IgA may mediate control of bacterial burden through both FcαR dependent and independent mechanisms.

### Glycosylation Patterns in *Mycobacterium tuberculosis*

Going beyond subclass and isotypes, post translational modifications of antibodies such as glycosylation are linked to inflammation. Antibody glycosylation patterns can be applied to TB in order to distinguish latent from active TB. In the area of autoimmunity, glycosylation of IgG has been extensively studied as a biomarker of disease severity. Agalactosylated IgG is considered pro-inflammatory and is a prominent biomarker in rheumatoid arthritis where increases in agalactosylated IgG with fucose is associated with increased disease severity (135). In inflammatory bowel disease where substantial granulomatous T cell pathology occurs, agalactosylation of IgG increases with greater T cell-mediated tissue damage and elevated C-Reactive Protein (136). On the other hand, the addition of sialic acid on IgG appears to transform it from a pro-inflammatory to anti-inflammatory entity (137).

In two independent and geographically distinct human cohorts, the active TB antibody glycome of total IgG exhibits agalactosylation and less sialic acid, but more fucose compared to latent TB IgG (26). While the lack of galactose is associated with inflammation in general, more specifically, agalactosylated IgG has been hypothesized to activate the mannose binding lectin complement pathway (69). This would be consistent with blood transcriptional profiling demonstrating elevated complement levels in individuals with active TB (138). The presence of sialic acid is associated with an anti-inflammatory state in rheumatological diseases (135), and this would be consistent with less inflammation in the context of latent compared to active TB (26). Antibodies with less fucose are better at engaging FcγRIIIa on NK cells to mediate ADCC in the monoclonal antibody literature (71, 139). Lu et al. did find higher binding of polyclonal IgG to FcγRIIIa along with enhanced TB-specific ADCC in latent compared to active TB (26). Intriguingly, an independent study of multiple human cohorts of latent and active TB demonstrated an NK cell signature associated with latent infection. This study also observed that PBMCs from latent TB individuals had increased capacity to mediated ADCC responses compared to uninfected controls, although this same analysis was not conducted using PBMCs from active TB individuals (140). Further studies will be important in orthogonally validating the link between specific IgG glycan patterns and Fc effector functions in human TB infection.

In contrast to IgG, the glycosylation of pentameric IgM is far more complex (141, 142) and therefore less well-characterized and understood. In a mouse model consistent with active TB, IgM glycosylation in BCG naïve mice after *Mtb* infection had a 5-fold increase in fucosylated IgM as well as less sialylation (143). Overall, mice receiving BCG had less fucosylation (143). What this means for downstream effector functions is less clear. Moreover, the respective similarities and differences between mice and human glycosylation regulation are less well-described. Thus, IgM glycosylation within and even beyond TB is an area that would benefit from significant further studies.

### Antibody Function in *Mycobacterium tuberculosis*

With differential glycosylation, isotypes, and subclasses, antibody Fc-domain engagement of Fc receptors on immune cells help modulate pro- and anti- inflammatory signals, the balance of which, in TB, can contribute to outcome (79). Despite lower *Mtb*-specific IgG titers in latent compared to active TB, there is higher affinity for the activating FcγRIIIa and no difference in the activating FcγRIIa or inhibitory FcγRIIb (26). Studies in mice deficient of the inhibitory FcγRIIb have greater control of *Mtb* bacterial burden, whereas complete knock-out of the Fcγ-chain region, which is essential for activating FcγR signaling, resulted in more severe disease in pulmonary pathology (144). Additionally, there are higher levels of FcγRI receptor gene expression in individuals with active TB compared to latent TB (145), and these levels decrease over the course of therapy (146). FcγRI is an activating receptor, which is upregulated by cytokines such as IFN-γ and GM-CSF and binds with high affinity to IgG1, IgG3, and IgG4 in comparison to the low affinity FcγRIIIa or IIa (79). As such, high FcγRI levels in active TB may either be a marker of or contribute to the inflammation. These lines of evidence at an Fc receptor level suggest that while too much immune complex activity identified in active TB may contribute to inflammation and pathology, some antibody mediated cellular effector functions may be important for bacterial control.

At a cellular level, immune cells expressing Fc-receptors have been implicated in both the enhancement of bacterial uptake, as well as the control of bacterial fate, in the context of antibodies. Early studies by Fong et al. (147) and Armstrong and Hart (148) coated *Mtb* with serum from BCG immunized rabbits. Though there was enhancement of uptake *in vitro* demonstrated by Armstrong and Hart, there were no significant difference in intracellular *Mtb* growth in rabbit (147) or murine (148) peritoneal macrophages. In humans, THP-1 monocytes demonstrated enhancement of BCG opsinophagocytosis using human serum after vaccination, inducing a humoral response that included IgG reactive to arabinomannan, a component of mycobacterial cell wall (149). This enhanced microbial uptake can be extended to primary human neutrophils, monocytes, and macrophages and attributed for the most part to IgG by depletion assays (150). Importantly, unlike Fong et al. and Armstrong and Hart, when both Chen et al. and De Valliere et al. examined the resulting *in vitro* bacterial burden, there was decreased growth rate of BCG when opsonized with post- compared to pre- vaccination serum in both neutrophils and monocytes/macrophages. Demonstrating that despite increased initial uptake of BCG, the fate of these bacteria surrounded by antigen-specific antibodies inside a cell was death. Whether differences in mycobacterial species (*Mtb* vs. BCG) with partially overlapping antigenic repertoires and/or host species FcR repertoires (rabbit vs. human vs. mouse) explains these differential findings, remains to be clarified. However, the more recent studies using human serum with human cells may provide a path toward overcoming species differences in modeling *Mtb in vitro*, particularly in the context of antibodies.

In studies with human cells and *Mtb*, macrophages, NK cells, and alveolar epithelial cells have been demonstrated to both potentially inhibit and also enhance bacterial growth in the context of antibodies. Opsonization of *Mtb* with polyclonal IgG purified from individuals with latent and active TB appeared to lead to similar bacterial uptake in primary human monocyte derived macrophages (26). However, upon the addition of purified polyclonal IgG after *Mtb* infection, intracellular bacterial burden is decreased in the context of antibodies from latent compared to active TB individuals (26). Whether or not this is due to cis- or trans- ADCC mediated by bystander macrophages in place of canonical NK cells remains unclear, but could provide a scenario in which this cellular effector mechanism (which is noted to be increased with *Mtb* antigen coated beads and NK cells in the context of latent compared to active TB IgG) could be directly linked (26). Finally, using arabinomannan reactive monoclonal antibodies to opsonize *Mtb*, Zimmerman et al. demonstrated that IgG1 on THP1 monocytes had no significant difference on bacterial counts compared to isotype control whereas the IgA1 isotype did (27). Indeed, when this experimental setup was expanded to the A549 human alveolar epithelial cell line expressing FcRn but not FcγRI, FcγRIII, FcγRII, or FcαR there was an enhancement of CFU noted upon infection with *Mtb* opsonized with monoclonal IgG isotype and inhibition with the IgA isotype. These studies demonstrate that bacterial fate is determined by both the antibody itself as well as the host cell.

A plethora of FcR expressing cells and associated downstream cellular processes can be recruited by *Mtb* reactive antibodies. Specifically, the classical antibody elicited effector functions of NK cell activation and subsequent production of granulysin to mediated ADCC has been observed (26, 140, 151). Moreover, Lu et al. demonstrated that phagolysosomal fusion, inflammasome activation, and IL-1β production could be elicited more with polyclonal IgG purified from individuals with latent compared to active TB. This is consistent with monoclonal antibodies enhancing BCG lysosomal co-localization (152). However, additional macrophage-mediated cellular processes in the context of *Mtb* include the production of additional cytokines, antimicrobial peptides, and even autophagy. These specific processes in mice and humans can contribute to protection as well as pathology (153, 154). Thus, even if these cellular functions are linked to antibodies, the implications for overall disease and outcome are not straight forward. These nuances may in fact help explain how enhanced survival (155) and or protection against dissemination (156) can be mediated by some monoclonal antibodies without impact on bacterial burden itself, highlighting further the immunomodulation and inflammatory balance that may be critical for TB control.

## Conditions Associated With TB Reactivation: HIV-1 Infection

### Inflammation in HIV-1

In acute HIV-1 infection the viral load rapidly increases alongside a burst of cytokines such as IFN-α and overstimulated pro-apoptotic CD4+ T cells; the immunological hallmark of HIV-1 infection (157). There is mounting evidence that chronic HIV-1 disease progression is characterized by systemic immune activation and oxidative stress, which can occur even while viremia is controlled with antiretrovirals (158–160). This systemic inflammation is thought to be caused by a number of factors; namely gut permeability, activated monocytes and cytokine derangement (160–163). High levels of viremia are accompanied by high levels of IFN-γ, TNF-α, IFN-α, and other inflammatory markers (164–166). The elevation of inflammatory serum markers is also correlated with onset of AIDS, cardiovascular disease, and lymphoma (165, 167–169).

### Antibody Isotypes and Subclasses in HIV-1

Recent studies showing the importance of non-neutralizing antibodies in HIV-1 control and in protection have expanded the framework of antibodies beyond direct neutralization (170). These antibodies have been relatively well-characterized in HIV-1, with momentum gaining after the RV144 HIV-1 vaccine immune correlates analysis showed non-neutralizing V1V2 HIV-1 Envelope (Env) IgG was associated with protection (171–174). Antibody responses in the first months of HIV-1 infection are governed by non-neutralizing antibodies directed against Env (175). While initially thought to be ineffective, these early antibodies display some viral inhibition, while higher ADCC activity of vaccine-induced IgG has been associated with reduced peak viral load in macaque SHIV trials (176–178). The isotypes that predominate during this time are IgM followed by class-switching to IgG and IgA (179, 180). Anti-Env IgM titers decline after 1 month of infection while anti-env IgG levels remain raised and neutralizing IgG titers emerge months to years later in infection (181, 182). The role of IgA in HIV is controversial. Vaccine-specific serum IgA was correlated with reduce vaccine efficacy in the RV144 trial, as did an elevated IgA:IgG ratio (171, 183). Further, serum IgA has been associated with HIV disease progression (184, 185). However, mucosal IgA may be functionally distinct compared to serum IgA, and there are promising results from non-human primate SHIV infection studies, where mucosal administration of IgA appears to be protective (186).

During acute infection, IgG3 is elicited first in the IgG subclass response but eventually is surpassed by higher IgG1 titers against Env (187). Multiple studies have associated HIV-specific IgG3 with viral control and enhanced Fc-functions (172, 178, 188, 189). IgG1 and IgG2 against internal HIV-1 p24 protein have also associated with viral control though the mechanisms are still not understood (190, 191). As with active TB, IgG1, and IgG3 subclass predominates in HIV-1, however the connection between these specific IgG subclasses and inflammation in the context of HIV-1 is yet to be investigated. Studies have identified a greater breadth of IgG neutralizing antibodies in HIV controllers who had high inflammatory markers such as TNF-α (192). Given the observation that neutralization breadth is a product of persistent viral replication (182), the high inflammatory markers in this study may point to a lack of viral control. In the context of HIV-1, some inflammation may be somewhat beneficial with cytokines and stimulated T cells encouraging the rate of somatic hypermutation in B cells to produce cross-reactive antibodies. However, in the context of a co-infected patient with latent TB, one could hypothesize that this HIV-associated inflammation involving inflammatory cytokines and T cell activation may tip the inflammatory balance too far, encouraging *Mtb* replication and development of active TB.

### Glycosylation of Antibodies in HIV-1

Glycosylation of antibodies in HIV-1 infection differ between bulk IgG and HIV-specific IgG (193). Chronic progressive HIV infection exhibits highly agalactosylated IgG compared to controls, which as previously discussed, is typical of inflammation. In studies of HIV-1 glycosylation, this highly agalactosylated IgG has been shown to engage the complement cascade (193–195). The subclass that exhibits most agalactosylation in HIV-1 infection was IgG1 followed by IgG2 and IgG4 (194). The ability to categorize IgG1 as protective or destructive may rely on its galactosylation status and subsequent impact on downstream inflammatory outcomes such as tissue destruction and innate cell activation via FcγRs. The role of sialylation and fucosylation in HIV-1 is not as clear. A recent study observed decreased sialylation and increased fucosylation of IgG in HIV-infected individuals (195). This increase in fucosylation was associated with reduced ADCC activity, meaning beneficial innate responses were decreased thereby reducing anti-inflammatory activities. Further, a significant association was observed between anti-inflammatory sialylated IgG, higher CD4+ counts and lower T cell activation (195). One caveat of this study is that they assessed the total IgG glycome and not HIV-specific IgG, meaning that we cannot comment on how specific IgG glycosylation impacts the ability of the antibody to fight HIV. While the global IgG glycome is an antibody inflammatory biomarker reflecting activated immune cells and therefore influencing an individual's susceptibility to disease, the glycosylation patterns of those specific antibodies which neutralize or function with ADCC/ADP to fight disease is also an important measure of the targeted immune response.

### Antibody Function in HIV-1

Multiple studies have demonstrated the importance of antibody Fc function in HIV-1 control and vaccine-mediated protection (196–200). ADCC responses to Env have been associated with improved clinical outcomes and places mutation pressure on HIV-1 virus (196, 199, 201–203). While historically HIV studies have focused upon NK cell mediated ADCC, recent research has demonstrated the importance of other Fc functions mediated by neutrophils, macrophages, monocytes, and complement activation (204, 205), such that polyfunctional antibody responses may be beneficial (172, 206, 207). Through the strong engagement of these particular Fc receptors in HIV infection, cells are activated resulting in downstream release of inflammatory cytokines and cytotoxic granules. A study looking at cytokines before pre- and post-ART (anti-retroviral therapy) in individuals with HIV-infection alone, found that TNF-α and IL-6 (pro-inflammatory) and IL-10, IL-4, and TGF-β (anti-inflammatory) were higher before ART while IFN-γ levels were lower before ART (208). In a different study of ART-naive individuals co-infected with HIV/TB, *Mtb* culture-positive status correlated with high TNF-α, IL-2, IL-12, and also IFN-γ (209). It may be possible, that those with HIV-infection and substantial TB reactivation risk exhibit an over-inflammatory state involving more pro-inflammatory cytokines such as IFN-γ, but the involvement of antibodies in this inflammation is yet to be studied.

### HIV-1 and *Mycobacterium tuberculosis* Co-infection

Infection with HIV-1 is a major risk factor for progression from latent to active TB, with the risk of reactivation 20-fold higher than the general population (158). The risk of developing active TB infection doubles in the first year of HIV-1 infection (210). The lack of T cells in HIV infection is one reason for the increased risk of TB acquisition and progression to active disease. However, even with effective ART such that viral loads are suppressed, CD4+ counts are >500 cells/μL and opportunistic infection risk is normalized, the risk of TB acquisition or reactivation is still increased (211). One study in South Africa, found that despite the widespread roll-out of ART, HIV prevalence among TB patients remained at 49% (212). Bucşan et al. recently found in a SIV/TB co-infection model that depleting CD4+ T cells in macaques was not sufficient to induce latent TB reactivation (213). The group propose SIV-associated reactivation may involve inflammatory imbalance. This could be due, in part, to inflammatory antibodies in HIV infection. Regardless, there may be other immune mechanisms than CD4+ T cell reduction that contribute to HIV-associated TB reactivation.

HIV-1 perturbs the *Mtb* immune response by impacting granuloma formation, *Mtb*-specific T cell response and macrophage activity (138). In an SIV-*Mtb* co-infection model, latent TB reactivated within 47 weeks of uncontrolled SIV infection (214). Immune Reconstitution Inflammatory Syndrome (IRIS), is a syndrome marked by innate cell cytotoxicity and cytokine dysregulation typically following the commencement of ART in HIV-1 infection. In IRIS, as the function of immune cells is reinstated, excessive inflammation and inflammatory driven organ-damage ensues. TB associated reactivation with IRIS can occur within 8 weeks of ART being commenced, particularly in the setting of advanced HIV-1 infection, indicating the role that this inflammatory state may play in TB reactivation (215–217). Further, *Mtb* and HIV co-infection increases the incidence of IRIS by 2-fold and HIV-infected patients with IRIS are at risk of acquiring TB (218, 219). The “unmasking” of other dormant pathogens, has also been observed in this hyperinflammatory state (220). The role of antibodies in the reactivation of dormant pathogens such as *Mtb* in the context of inflammation must be further explored.

### Antibodies in TB/HIV-1 Co-infection

While IgG is formed against *Mtb* antigens in patients co-infected with *Mtb* and HIV-1, some studies observe substantially lower titers of *Mtb*-specific IgG and other isotypes (221, 222). Yu et al. (222) show correlations between IgG2 and IgG3 as well as IgG1 and IgG3 levels in HIV/TB co-infection (222). The group consider HIV-associated hypergammaglobulinemia as a cause of the IgG correlations and comment that the TB serology they assessed is more complicated in HIV negative individuals than in HIV/TB co-infected patients. It is pertinent to consider the impacts of losing T-cell help on isotype and subclass switching in the context of this HIV co-infection.

In contrast to these findings, other groups have found an increase in *Mtb*-specific antibody titers and reactivity in HIV/TB co-infection which they attribute to a high *Mtb* bacillary burden in HIV infected individuals (223). Yu et al. also highlight in their discussion, the counterintuitive nature of TB antibody and IFN-γ research, in that some show them to be protective, but others find that high antibody and IFN levels correlate with bacterial burden. The authors warn against speculating whether specific immune mechanisms can be concluded from their own study. One could hypothesize that some level of bacterial load is necessary for the instigation of an immune response but if that replication is overbearing, infection cannot be controlled.

Studies into the breadth of the antibody response in HIV/TB co-infection are limited. One recent study proposes the measurement of serum LAM as a biomarker for active TB (224) and Yu et al. (222) assessed *Mtb* polysaccharide responses and found elevated arabinomannan-specific IgG2 had the highest titers in all TB-infected groups. The fact that different subclasses predominate based on the antigen type is unsurprising as IgG2 is predominately generated against polysaccharide antigens. This does highlight the complexity of *Mtb* being both bacterial (generating typically a Th2 response) and an intracellular pathogen (needing Th1 responses too) and informs us as to why the ability to delineate protective vs. pathogenic immune responses to *Mtb* are so difficult to characterize.

*Mtb*-specific antibody titers decrease with HIV/TB co-infection as does the binding avidity of these antibodies to *Mtb* targets such as Ag85A (38). This group attribute the reduced avidity to dampened B cell responses, possibly caused by reduced T cell help that occurs with HIV infection, and the reduction in avidity means the *Mtb*-specific antibody responses are not as effective. It is important to investigate whether these ineffective antibody responses may contribute to a lack of *Mtb* control in HIV/TB co-infection. One genomics study found the deletion of FcγRIIIb genes was higher in patients co-infected with HIV and TB compared to HIV-1 alone (225). FcγRIIIb is a low-affinity activating receptor only expressed on neutrophils and basophils (79). The effects of this gene deletion on downstream receptor-expression and antibody function in co-infection merits further investigation. Regarding glycosylation in co-infection, a study of HIV-1 infected individuals with *Mycobacterium avium* infection showed that co-infected individuals have higher levels of agalactosylated IgG as well as elevated serum IgA levels (226). Whether agalactosylation is also increased in HIV-1 co-infection with *Mtb* is yet to be studied. If future studies find that agalactosylated IgG are increased in HIV/TB co-infection, we would consider whether these inflammatory antibodies are involved in TB reactivation by enhancing a state of over-inflammation.

## Conditions Associated With TB Reactivation: Diabetes Mellitus

### Diabetes Mellitus and Inflammation

Type II diabetes mellitus (T2DM) is typically caused by nutritional, lifestyle, and genetic factors causing obesity-induced insulin resistance and eventual depletion of insulin-secreting cells altogether. T2DM is characterized by a proinflammatory milieu with CD8+ T cell and T_H_1 activity promoting insulin resistance and glucose intolerance (227–229). High-fat diets contribute to inflammation in diabetes and obesity in that they supply high levels of fatty acids which stimulate immune receptors such as toll-like receptors on innate cells and non-immune cells in adipose tissue. This cell activation encourages secretion of pro-inflammatory cytokines and skews the activation state of immune cells such as macrophages and B cells to one of inflammation. This pro-inflammatory milieu includes high neutrophil activation, macrophage adipose and pancreatic infiltration, increased pro-inflammatory cytokines such as IL-1β, IL-8, and TNF as well as decreased anti-inflammatory cytokines such as IL-10 (230–233). IL-10 typically inhibits activity of T_H_1 cells, macrophages and NK cells as well as regulating class-switching of B cells to IgM and IgG plasmablasts (234, 235).

Macrophage and CD4+ T cell activation in T2DM pathogenesis is well-described (236, 237). Studies show upregulation of T_H_1 cells in T2DM adipose tissue with pro-inflammatory signatures (238). Not dissimilar to active TB, increases in TNF-α secretion by T_H_1 and T_H_17 CD4+ T cells are attributed to T2DM pathogenesis (239). One recurring cell-type in diseases where TB reactivation is high, are macrophages and monocytes. The immune environment of high TNF increases monocyte infiltration and IL-1, IL-6, and IL-12 are associated with macrophage and monocyte activation (240). With these two cell types being the primary targets of *Mtb* infection, it could be proposed that the induction of this macrophage-driven inflammation in some diseases increases TB reactivation risk, providing an environment within which *Mtb* can replicate. The complexity of the macrophage-TB response has been detailed by Guirado et al. in the following review (241).

### Antibodies Isotypes in Diabetes Mellitus

The involvement of antibodies in T2DM pathogenesis is not well-understood. One study in patients with T2DM found a smaller percentage of antigen-specific antibody secreting plasmablasts, but when stimulated with LPS, T2DM B cells produced greater IgM and IgG titers (233). There were no differences in IgA titers to healthy controls. Further, the B cell phenotype in T2DM is similar to that found in obese individuals, yet those with T2DM failed to elicit protective B cell responses to an influenza vaccine while obese individuals did elicit protective responses (233). This may indicate that in the proinflammatory environment, T2DM patients have readily activated B cells producing antibody isotypes IgG and IgM, however that these antibodies may be undirected or enhance disease, counteracting any protective neutralization effects seen otherwise. IgG has also been implicated in its association and specificity to long chain saturated fatty acids found in the serum of T2DM patients (242).

High fat diets are associated with systemic immune activation and the secretion of “pathogenic” IgG which promotes insulin resistance (243). This study transferred IgG from mice that received a high fat diet into recipient mice with a normal diet and found that post-transfer, recipient mice had elevated TNF-α and “pathogenic” IgG which promoted glucose intolerance and insulin resistance via Fc engagement (243). The authors discuss phenotypic changes to macrophages and B cells in adipose tissue that results in inflammatory cytokine release and “pathogenic” IgG.

Further, an IgG autoantibody was associated with T2DM prevalence and earlier disease onset in a Southwest American Indian cohort (244). It is thought that IgG antibodies form immune complexes in visceral adipose tissue and engage FcγR on B cells and macrophages, inducing inflammatory cytokines (243, 245). Pathological autoantibodies exist in other autoimmune diseases such as systemic lupus erythematous where autoantibody subclasses in the order IgG1>IgG3>IgG2 are substantially elevated (246, 247). There are case reports of TB infection in autoimmune affected individuals, however this review does not discuss these cases as immunological data lacks in these cohorts (248). Looking at the role of IgG subclasses in T2DM, total IgG2 has been associated with insulin-resistance in mouse skeletal muscle through activation of epithelial FcγRIIb, an inhibitory Fc-receptor, and the impairment of insulin epithelial translocation (243, 249, 250). For endothelial cells, since FcγRIIb is engaged in insulin translocation it is necessary to maintain insulin sensitivity as shown by the attenuation of FcγRIIb resulting in impaired insulin translocation in skeletal muscle of obese mice (250). With elevated levels of IgG2 binding to FcγRIIb, insulin translocation is impaired. While this FcR-binding mechanism may not directly increase inflammation, it may be a by-product of an overactive immune system which encourages the over-production of “pathogenic” IgG.

### Antibody Glycosylation and Function in Diabetes Mellitus

The first large study on antibody N-glycans in T2DM patients found that T2DM IgG derived glycans had substantially increased agalactosylation and decreased sialylation of all fucosylated structures (251). The authors refer to their findings as “reflective of an overall pro-inflammatory state” (251). The implications of these findings are that T2DM IgG engage more readily with the complement cascade, bind with higher affinity to FcγR and impact insulin resistance. Further, this study found increased fucosylation of IgG structures with bisecting GlcNAc, and decreased fucosylation of IgG glycans without bisecting GlcNAc in T2DM. The glycosylation of antigen-specific antibodies in T2DM is not well-understood and would merit from further investigation as to elucidate whether they mechanistically cause inflammation. In mice, reduced N-glycan IgG sialylation was associated with insulin resistance and glucose intolerance as associated with a T2DM phenotype (250). When IgG from T2DM humans was transferred into B-cell deficient mice, insulin resistance developed. Interestingly, this obesity-related insulin resistance and glucose intolerance could be reversed by normalizing the sialylation patterns of IgG. Further, C-reactive protein (CRP), a marker of inflammation in T2DM, caused insulin resistance in this same study. They also determined that CRP acted on the inhibitory receptor FcγRIIb present on epithelial cells, stopping translocation of insulin through the epithelial membrane (250). Mice that were FcγRIIb-knockout, were protected from insulin resistance.

In the chronic hyperglycemic state of diabetes, there is another type of glycosylation which can occur. This process is called non-enzymatic glycosylation of plasma proteins which occurs when plasma is saturated with glucose due to hyperglycemia and attaches spontaneously to plasma proteins. Unlike N-linked glycans, non-enzymatic glycosylation has been well-documented in diabetes and other diseases for many years (252). Non-enzymatic glycosylation of IgG has been implicated in diabetes pathogenesis affecting renal clearance of IgG with subsequent effects on renal function. Further, glycosylated IgG has been suggested as a comparable biomarker to the well-known glycosylated hemoglobin (HbA1c) (253, 254).

### Antibodies in Diabetes Mellitus and Tuberculosis Comorbidity

Diabetes with associated *Mtb* infection is a substantial global health issue. There are now more individuals comorbid with TB/Diabetes than those with TB/HIV-1 (14). Diabetics with associated pulmonary TB infection have higher levels of type-1 pro-inflammatory cytokines such as IFN-γ and TNF-α as well as a reduction in anti-inflammatory cytokine IL-22 (255). The elevation of IFN-γ and TNF-α and reduction in anti-inflammatory cytokines mean that inflammatory pathways in innate cells and T cells are discharged which contributes to immunopathology and poor *Mtb* control in diabetic individuals (255). Further, T2DM co-infected with TB have a pro-atherogenic, dyslipidaemic plasma profile exacerbating metabolic disease which is postulated to contribute to TB reactivation (256). There are a number of studies that characterize the DM/TB co-infection T cell and innate cell phenotypes but none include a characterization of antibody responses in DM/TB co-infection (257–259). A study by Gomez et al. (260) demonstrated that monocytes cultured with serum from diabetics do not associate via classical or alternative complement pathways with *Mtb in vitro*, and they attribute this to reduced FcγR and complement receptor defects (260, 261). Defective FcγR would impact IgG isotype binding and functionality. The group do not report antibody nor complement protein titers in these individuals which would have a addressed the question of whether reduced antibody titer in diabetics is a potential cause for the lack of *Mtb* association. Given the increasing burden of DM/TB co-infection, urgent attention needs to be given to potential immune biomarkers of reactivation, such as antibodies.

## Conditions Associated With TB Reactivation: Chronic Kidney Disease

### Chronic Kidney Disease and Inflammation

Chronic Kidney Disease (CKD) and its further progressed End-Stage Renal Disease (ESRD) are associated with substantial immune perturbation and inflammation (262). Despite the varied etiology of CKD, the end point of inflammation encompassing renal impairment, metabolic waste accumulation and treatment with dialysis is universal to CKD patients. The impact of hemodialysis includes chronic activation of the alternative complement pathway, antigenic stimulation and disruption of mucocutaneous barriers (263). CKD is a systemic disease not only affecting the kidneys, with “uremia” affecting other organs including the lungs. Pro-inflammatory cytokines such as TNF-α, IFN-γ, and IL-6 are elevated while IL-10 levels are suppressed (262, 264). Further, T_H_1 responses are elevated in ESRD and Kato et al. discuss this immune dysfunction in ESRD in more detail (264). Impaired immune function in CKD leads to higher rates of infection while the exaggerated inflammatory immune response in the condition contributes to different immune-related events such as atherosclerotic cardiovascular disease (262). This is thought to occur in HIV-1 and T2DM also. It may be possible that the dysregulated immunity with excessive IFN-γ, TNF-α, and T_H_1 responses, promotes macrophage activation and contributes to an environment which may favor *Mtb* replication.

### Antibody Isotypes and Subclasses in Chronic Kidney Disease

A number of studies have documented reduced titers of antigen-specific IgG, IgA, and IgM in patients with chronic renal insufficiency. Most in-depth serum antibody analyses involve antigen-specific responses to vaccination or infection in CKD cohorts. The antibody response to Hepatitis B vaccine is muted in CKD individuals, with low seroconversion and rapid reduction in titers post-vaccination (265). Further, in Hemophilus influenza A-vaccinated CKD patients, there are reduced absolute B cell numbers and less functional bactericidal antigen-specific IgG and IgM (266). In a recent study, aiming to elucidate the role of the T-cell inhibitory receptor CTLA-4 in pathogenesis of glomerulonephritis, a major cause of CKD, levels of IgM inversely correlated with lower CTLA-4, as did IgG and IgA to a lesser extent, with the proposition that this may reflect inappropriate IgM function in ESRD (267). Further, many causes of CKD are auto-immune based and involve the generation of autoantibodies against kidney tissue. These auto-antibodies tend to be of the subclasses IgG1 and IgG3 and are reviewed at a greater depth by Tecklenborg et al. (268).

### Glycosylation and Function of Antibodies in Chronic Kidney Disease

There is very little data on glycosylation of antibodies in CKD. The most recent study found a predominance of pro-inflammatory agalactosylated IgG Fc-glycosylation in patients with CKD and in those with agalactosylated IgG but no CKD, they were at higher risk of developing CKD (269). Further, IgG Fc N-linked glycans in kidney disease patients are more likely to lack a core-fucose, a phenotype associated with substantially increased FcγRIIIa binding affinity and ADCC. Individuals with sialylated, core-fucosylated with bisecting GlcNAc IgG had greater CKD risk. This group also found that levels of anti-inflammatory IgG Fc-glycan sialylation were decreased in patients with CKD (269). One group also discovered a γ-chain deficiency in mice, thereby preventing the function of activating FcγRs, resulting in kidney disease protection (270). These findings amongst those on glycosylation in CKD, are consistent with the idea that CKD progression is linked to pro-inflammatory mechanisms, some of which are enacted by antibodies. Anti-neutrophil cytoplasmic autoantibody vasculitis is one cause of CKD and Fc N-glycan patterns of IgG1 in this disease has also been described as pro-inflammatory, with high agalactosylation and low sialylation patterns (271).

### Antibodies and Immunity in Chronic Kidney Disease and Tuberculosis Comorbidity

Despite notably high rates of co-infection between TB and CKD, the diagnosis of latent TB is difficult in this population. This is due to lowered sensitivity of the IGRA assay in detecting *Mtb* in ESRD groups which has been attributed to defective T cell function (272). γδ T cells in this co-infected population showed lowered expression of lung-homing receptors while MAIT cells were depleted in the periphery, with particular reductions in cells also associated with lung-trafficking (273, 274). This indicates a potential role for unconventional T cells in controlling pulmonary TB infection. However, very little research has looked into characterizing antibody profiles in patients comorbid with kidney disease and TB. This is to the exception of IgA-nephropathy, a leading cause of primary kidney disease which is due to deposition of IgA in renal tubules leading to glomerulonephritic disease and eventually CKD. In a cohort of IgA-nephropathic patients co-infected with TB, levels of anti-Ag85A antibody were higher in co-infected patients indicating an ability to generate *Mtb*-specific antibody (275). It is thought that elevated *Mtb*-specific IgA levels contribute to IgA nephropathy co-infection, glomerulonephritis and ensuing CKD (276). The pathogenesis of this involves alternative complement activation, lectin activation and deposition of IgA-complexes in the kidney (275, 276). Deposits of IgA1 are found at higher levels of co-infected individuals as well as patients presenting with renal TB (275). Further, high levels of TGF-β1 are correlated with defective IgA1 generation and TGF-β1 increases in both TB and IgA nephropathy (276). These papers together propose that *Mtb* infection increases the levels of IgA in serum which aggregate to form pathogenic complexes leading to nephritic disease. This then exacerbates kidney disease and the cycle of inflammation, which may worsen TB disease. More than that, it may be possible that these serum IgA1 antibodies act more readily on inflammation in the kidney hence the association with higher TB renal disease in this population.

Studies assessing *Mtb* proteins and kidney injury found the *Mtb* antigen, ESAT-6, contributed to renal injury in mice injected with a high dose of ESAT-6 by upregulating the expression of pro-inflammatory microRNA-155 via the MyD88 innate cell pathway (275, 277, 278). While the dose of ESAT-6 used in these studies may not reflect those found in physiological disease, this evidence may show a cyclical worsening of disease in CKD/TB coinfection exacerbated by kidney inflammation caused by TB and CKD independently.

TB mortality is also very high amongst ESRD patients after kidney transplant. A recent retrospective analysis discovered that kidney transplant recipients receiving the immunotherapy Belatacept, a fusion protein of anti-CTLA-4 and the Fc-portion of IgG1 (CTLA4-Ig), had a significantly higher incidence of TB reactivation and infection (279). The incidence of TB in the group receiving Belatacept was higher at 17.6% compared to other immunosuppressive drugs where the incidence was <2.5% indicating that the immunosuppressive actions of these drugs may not have been the root cause for TB reactivation in these groups (279). This is interesting given the previous studies discussed above provide some evidence to support the proinflammatory actions of IgG1 may worsen TB disease prognosis. Belatacept or CTLA4-Ig, was developed to not only stimulate T cells through inhibiting CTLA-4, but to induce ADCC and complement-dependent cytotoxicity via the IgG1 Fc region (280). The findings of TB reactivation in this anti-CTLA4 immunotherapy group echo the recent case reports of TB reactivation with PD-1 inhibitors (96, 98, 99, 281). Exaggerated T_H_1 responses due to PD-1 inhibitors have been proposed as the mechanism by which TB reactivates in these patients (99).

## Conclusions

*Mtb* infection and its frequent association with HIV, T2DM, and CKD are growing global health burdens. With a quarter of the world latently infected with *Mtb* and the rise of non-communicable diseases such as T2DM and CKD in both developed and developing countries, we must pay attention to this double burden of disease. There is a notable lack of research on the mechanisms of TB reactivation in different immune environments. The paradoxical evidence around T cell responses and type 1 cytokines suggests that high IFN-γ, TNF-α, and IL-2 CD4+ T cells induced in response to vaccination or before the establishment of TB disease is beneficial to TB control, but when these responses are too late or they expand after *Mtb* has established, they are detrimental. A better understanding of TB reactivation would allow the rational design of preventative measures to reduce associated mortality and burden on individuals and their healthcare systems. In this review, we describe antibody characteristics of isotypes and subclasses, glycosylation patterns and function across TB, HIV-1, T2DM, and CKD (see summary in Supplementary Table 1). There is a large volume of evidence from both arms of the immune system that point to the role of a pro-inflammatory milieu in assisting TB reactivation. HIV-1, T2DM, and CKD are highly inflammatory chronic diseases, and this is reflected by their antibody profiles, especially IgG subclasses and post-translational modifications. However, clearly this review also highlights the many gaps in our knowledge. To genuinely understand the causes of TB reactivation, we must look at the interface at which antibodies, T cells and innate cells all interact to create an inflammatory response. Much can be learnt from the actions and patterns of antibodies from these diseases. Furthermore, antibodies are a real-time measure of immunocompetency and assessing serum antibodies is comparatively more reliable, cost-effective and high-throughput in comparison to cell-based assays. Understanding antibody characteristics is essential to decode these patterns in disease, and we are gaining in momentum and understanding.

## Author Contributions

MM and AC contributed to conception of the review. MM compiled literature, wrote and edited drafts, and created figures. MM, LL, AC, and SK contributed to the manuscript editing and revisions and approved the submitted version.

### Conflict of Interest

The authors declare that the research was conducted in the absence of any commercial or financial relationships that could be construed as a potential conflict of interest.
