# Supplementary material for: An Inflammatory Story: Antibodies in Tuberculosis Comorbidities
Source: Front Immunol. 2019 Dec 9;10:2846. doi: 10.3389/fimmu.2019.02846 (PMC6913197; doi:10.3389/fimmu.2019.02846)
Supplement: Supplementary file 1 [file Table_1.DOCX]

|  | Global Burden | Isotype/subclass | Antibody Glycosylation | Antibody Functions |
| --- | --- | --- | --- | --- |
| Active TB | 10.4 million annual new infections (World Health Organization, 2018) | ↑ Increased IgG titers (Lenzini et al., 1977;Achkar et al., 2010;Lu et al., 2016;de Araujo et al., 2018)  - ↑ Increased IgG1>IgG3 titers  - ↑ Increased ESAT-6-specific IgG1  (Hussain et al., 1995;Sousa et al., 1998;Mattos et al., 2016)  IgG1-associated TNF-a release (Hussain et al., 2000)  Monoclonal *Mtb* IgG1 enhances bacterial replication in mouse model of active disease (Zimmermann et al., 2016)  IgA present in BAL from patients with pulmonary TB (Raja et al., 1988;Demkow et al., 2005)  Mice deficient in IgA had ↑ *Mycobacterium bovis* susceptibility (Rodriguez et al., 2005) | Active TB compared to latent TB:  - ↑ Increased IgG agalactosylation  - ↑ Increased IgG fucosylation  - ↓ Decreased IgG sialylation  (Lu et al., 2016)  Protective BCG caused ↓ IgM fucosylation following *Mtb* challenge in mice (Kumagai et al., 2019)  5-fold increase in IgM fucosylation and decreased sialylation in mouse model of active TB (Kumagai et al., 2019) | ↑ Increased FcγRI levels at time of active TB diagnosis  (Cliff et al., 2013)  ↑ Increased monocyte associated ADCP  (Lu et al., 2016)  ↑ Increased Monocyte:Lymphocyte ratio  (Zelmer et al., 2018)  Knock out of Fcγ-chain gene causes ↑ infection in mouse model of TB (Maglione et al., 2008)  ↑ Increased complement C1q levels greater than latent TB (Lubbers et al., 2018) |
| Latent TB | 1.7 billion infected (Houben and Dodd, 2016) | ↑ Increased IgG3 ratio in latency compared to active TB (de Araujo et al., 2018)  ↓ Decreased *Mtb* specific antibody titres compared to active TB (de Araujo et al., 2018) | Compared to active TB:  - ↑ Increased sialylation  - ↓ Decreased agalactosylation  (Lu et al., 2016) | ↑ Increased binding of polyclonal IgG to FcγRIIIa and NK cell-mediated ADCC compared to active TB  (Lu et al., 2016)  Better PBMC-mediated ADCC function in latent TB individuals (Roy Chowdhury et al., 2018) |
| HIV-1 | 37.9 million currently infected (UNAIDS, 2019) | Total V1V2 IgG associated with RV144 vaccine protection (Haynes et al., 2012;Zolla-Pazner et al., 2014)  V1V2 IgG3 associated with RV144 vaccine protection (Chung et al., 2014;Yates et al., 2014)  ↑ Increased Env-IgG3 increases viral control and Fc-function  (Dugast et al., 2014;Sadanand et al., 2018)  ↑ Increased Env-IgG3 in acute HIV-1 (Yates et al., 2011)  IgG1 > IgG3 in uncontrolled infection (Tomaras and Haynes, 2009)  ↑ Increased serum IgA associated with disease progression (Fling et al., 1988;Coates et al., 1992)  ↑ Increased serum Env-IgA (Haynes et al., 2012)  ↑ Increased IgA:IgG ratio associated with reduced vaccine efficacy (Tomaras et al., 2013) | ↑Increased agalactosylation of IgG1 > IgG2 > IgG4 in HIV-1 progressors (Moore et al., 2005;Ackerman et al., 2013;Vadrevu et al., 2018)  ↓ Decreased sialic acid in progressors  ↑ Increased fucose in progressors  (Vadrevu et al., 2018) | NK cell ADCC associated with Long Term Non-Progression/ HIV Controllers (Chung et al., 2011a;Chung et al., 2011b;Johansson et al., 2011;Wren et al., 2013)  Polyfunctional antibody responses associated with protection and HIV controllers (Chung et al., 2014;Ackerman et al., 2016) |
| HIV-TB | 1.2 million co-infections (World Health Organisation, 2018) | ↓ Decreased IgG titres (Sartain et al., 2006;Yu et al., 2012)  Correlation between IgG2 with IgG3 and IgG1 with IgG3 in HIV/TB compared to TB alone (Yu et al., 2012)  ↓ Decreased *Mtb*-specific antibody titres and ↓ binding avidity to Ag85A (*Mtb* antigen) (Kimuda et al., 2018)  ↑ Increased serum IgA (Hernandez-Munoz and Stanford, 1996) | ↑ Increased agalactosylation of IgG in HIV/*Mycobacterium avium* co-infection (Hernandez-Munoz and Stanford, 1996) | ↓ Decreased FcγRIIIb genes in HIV/TB compared to HIV alone (Machado et al., 2013) |
| Diabetes | 422 million people are insulin resistant (World Health Organization, 2016) | ↑ Increased LPS-induced IgG and IgM titres (Zhai et al., 2016)  ↑ Increased long chain saturated fatty-acid IgG in serum (Nicholas et al., 2015)  ↑ Increased ‘pathogenic’ IgG which promotes insulin resistance in mouse models (Winer et al., 2011)  Transfer of IgG from humans with T2DM into mice caused insulin resistance (Tanigaki et al., 2018) | - ↑ Increased agalactosylation and ↓ decreased sialylation of fucosylated IgG  - ↑ Increased fucosylation of bisected GlcNAc IgG  (Lemmers et al., 2017)  ↓ Decreased aialylated IgG associated with insulin resistance (Tanigaki et al., 2018)  ↑ Increased Non-enzymatically glycosylated IgG (Kalia et al., 2004;Mistry and Kalia, 2009) | Auto-IgG against adipose tissue engaged monocyte FcγR and complement (Mallat, 2011;Winer et al., 2011)  Mice with FcγRIIb-knock out are protected from insulin resistance (Winer et al., 2011;Fiorentino et al., 2018;Tanigaki et al., 2018) |
| Diabetes-TB | 4.57 million (Restrepo 2016) | Unknown | Unknown | Low association between diabetic monocytes and *Mtb* (Gomez et al., 2013*)* |
| CKD | 10 million deaths from kidney disease (Luyckx et al., 2018) | ↓ Decreased antibody response to Hep B vaccine (Bel'eed et al., 2002)  ↓ Decreased B cell IgG/IgM influenza responses in vaccinated patients (Gaultier et al., 2019)  ↑ Increased Auto-antibody IgG1/IgG3 (Tecklenborg et al., 2018) | - ↑ Increased agalactosylation  - ↓ Decreased sialylated IgG (Barrios et al., 2016)  IgG1 with ↑ agalactosylation and ↓ sialylation  (Lardinois et al., 2019) | γ-chain deficiency protected against kidney disease in mice (Lopez-Parra et al., 2012) |
| CKD-TB | 698 per 100,000 receiving dialysis (Romanowski et al., 2016) | ↓ Reduced IgA1 associated with ↑ increased TGF-beta (Wang and Tao, 2018)  ↑ Increased IgG1-based CTLA4-IgG associated with Mtb reactivation post kidney-transplant (Viana et al., 2019) | Unknown | ↑ Increased complement activation (Wang and Tao, 2018) |

**Supplementary Table 1.** Antibody characteristics in Active Mtb, Latent Mtb, HIV-1 and Mtb co-infection, T2DM and Mtb co-morbidity, CKD and Mtb co-morbidity and checkpoint blockage/Mtb reactivation.

REFERENCES FOR SUPPLEMENTARY TABLE 1.

Achkar, J.M., Jenny-Avital, E., Yu, X., Burger, S., Leibert, E., Bilder, P.W., Almo, S.C., Casadevall, A., and Laal, S. (2010). Antibodies against immunodominant antigens of Mycobacterium tuberculosis in subjects with suspected tuberculosis in the United States compared by HIV status. *Clin Vaccine Immunol* 17**,** 384-392.

Ackerman, M.E., Crispin, M., Yu, X., Baruah, K., Boesch, A.W., Harvey, D.J., Dugast, A.S., Heizen, E.L., Ercan, A., Choi, I., Streeck, H., Nigrovic, P.A., Bailey-Kellogg, C., Scanlan, C., and Alter, G. (2013). Natural variation in Fc glycosylation of HIV-specific antibodies impacts antiviral activity. *J Clin Invest* 123**,** 2183-2192.

Ackerman, M.E., Mikhailova, A., Brown, E.P., Dowell, K.G., Walker, B.D., Bailey-Kellogg, C., Suscovich, T.J., and Alter, G. (2016). Polyfunctional HIV-Specific Antibody Responses Are Associated with Spontaneous HIV Control. *PLoS Pathog* 12**,** e1005315.

Barrios, C., Zierer, J., Gudelj, I., Stambuk, J., Ugrina, I., Rodriguez, E., Soler, M.J., Pavic, T., Simurina, M., Keser, T., Pucic-Bakovic, M., Mangino, M., Pascual, J., Spector, T.D., Lauc, G., and Menni, C. (2016). Glycosylation Profile of IgG in Moderate Kidney Dysfunction. *J Am Soc Nephrol* 27**,** 933-941.

Bel'eed, K., Wright, M., Eadington, D., Farr, M., and Sellars, L. (2002). Vaccination against hepatitis B infection in patients with end stage renal disease. *Postgrad Med J* 78**,** 538-540.

Chung, A.W., Ghebremichael, M., Robinson, H., Brown, E., Choi, I., Lane, S., Dugast, A.S., Schoen, M.K., Rolland, M., Suscovich, T.J., Mahan, A.E., Liao, L., Streeck, H., Andrews, C., Rerks-Ngarm, S., Nitayaphan, S., De Souza, M.S., Kaewkungwal, J., Pitisuttithum, P., Francis, D., Michael, N.L., Kim, J.H., Bailey-Kellogg, C., Ackerman, M.E., and Alter, G. (2014). Polyfunctional Fc-effector profiles mediated by IgG subclass selection distinguish RV144 and VAX003 vaccines. *Sci Transl Med* 6**,** 228ra238.

Chung, A.W., Navis, M., Isitman, G., Centre, R., Finlayson, R., Bloch, M., Gelgor, L., Kelleher, A., Kent, S.J., and Stratov, I. (2011a). Activation of NK cells by ADCC responses during early HIV infection. *Viral Immunol* 24**,** 171-175.

Chung, A.W., Navis, M., Isitman, G., Wren, L., Silvers, J., Amin, J., Kent, S.J., and Stratov, I. (2011b). Activation of NK cells by ADCC antibodies and HIV disease progression. *J Acquir Immune Defic Syndr* 58**,** 127-131.

Cliff, J.M., Lee, J.S., Constantinou, N., Cho, J.E., Clark, T.G., Ronacher, K., King, E.C., Lukey, P.T., Duncan, K., Van Helden, P.D., Walzl, G., and Dockrell, H.M. (2013). Distinct phases of blood gene expression pattern through tuberculosis treatment reflect modulation of the humoral immune response. *J Infect Dis* 207**,** 18-29.

Coates, R.A., Farewell, V.T., Raboud, J., Read, S.E., Klein, M., Macfadden, D.K., Calzavara, L.M., Johnson, J.K., Fanning, M.M., and Shepherd, F.A. (1992). Using serial observations to identify predictors of progression to AIDS in the Toronto Sexual Contact Study. *J Clin Epidemiol* 45**,** 245-253.

De Araujo, L.S., Da Silva, N.B.M., Leung, J.a.M., Mello, F.C.Q., and Saad, M.H.F. (2018). IgG subclasses' response to a set of mycobacterial antigens in different stages of Mycobacterium tuberculosis infection. *Tuberculosis (Edinb)* 108**,** 70-76.

Demkow, U., Bialas-Chromiec, B., Filewska, M., Sobiecka, M., Kus, J., Szturmowicz, M., Zielonka, T., Augustynowicz-Kopec, E., Zwolska, Z., Wasik, M., and Rowinska-Zakrzewska, E. (2005). Humoral immune response against mycobacterial antigens in bronchoalveolar fluid from tuberculosis patients. *J Physiol Pharmacol* 56 Suppl 4**,** 79-84.

Dugast, A.S., Stamatatos, L., Tonelli, A., Suscovich, T.J., Licht, A.F., Mikell, I., Ackerman, M.E., Streeck, H., Klasse, P.J., Moore, J.P., and Alter, G. (2014). Independent evolution of Fc- and Fab-mediated HIV-1-specific antiviral antibody activity following acute infection. *Eur J Immunol* 44**,** 2925-2937.

Fiorentino, T.V., Succurro, E., Arturi, F., Giancotti, A., Peronace, C., Quirino, A., Sesti, F., Andreozzi, F., Hribal, M.L., Perticone, F., Foca, A., and Sesti, G. (2018). Serum IgG2 levels are specifically associated with whole-body insulin-mediated glucose disposal in non-diabetic offspring of type 2 diabetic individuals: a cross-sectional study. *Sci Rep* 8**,** 13616.

Fling, J.A., Fischer, J.R., Jr., Boswell, R.N., and Reid, M.J. (1988). The relationship of serum IgA concentration to human immunodeficiency virus (HIV) infection: a cross-sectional study of HIV-seropositive individuals detected by screening in the United States Air Force. *J Allergy Clin Immunol* 82**,** 965-970.

Gaultier, G.N., Mccready, W., and Ulanova, M. (2019). Natural immunity against Haemophilus influenzae type a and B-cell subpopulations in adult patients with severe chronic kidney disease. *Vaccine* 37**,** 3677-3684.

Gomez, D.I., Twahirwa, M., Schlesinger, L.S., and Restrepo, B.I. (2013). Reduced Mycobacterium tuberculosis association with monocytes from diabetes patients that have poor glucose control. *Tuberculosis (Edinb)* 93**,** 192-197.

Haynes, B.F., Gilbert, P.B., Mcelrath, M.J., Zolla-Pazner, S., Tomaras, G.D., Alam, S.M., Evans, D.T., Montefiori, D.C., Karnasuta, C., Sutthent, R., Liao, H.X., Devico, A.L., Lewis, G.K., Williams, C., Pinter, A., Fong, Y., Janes, H., Decamp, A., Huang, Y., Rao, M., Billings, E., Karasavvas, N., Robb, M.L., Ngauy, V., De Souza, M.S., Paris, R., Ferrari, G., Bailer, R.T., Soderberg, K.A., Andrews, C., Berman, P.W., Frahm, N., De Rosa, S.C., Alpert, M.D., Yates, N.L., Shen, X., Koup, R.A., Pitisuttithum, P., Kaewkungwal, J., Nitayaphan, S., Rerks-Ngarm, S., Michael, N.L., and Kim, J.H. (2012). Immune-correlates analysis of an HIV-1 vaccine efficacy trial. *N Engl J Med* 366**,** 1275-1286.

Hernandez-Munoz, H.E., and Stanford, J.L. (1996). IgA and IgG antibodies to distinct serotypes of Mycobacterium avium in HIV seropositivity and AIDS. *J Med Microbiol* 44**,** 165-169.

Houben, R.M., and Dodd, P.J. (2016). The Global Burden of Latent Tuberculosis Infection: A Re-estimation Using Mathematical Modelling. *PLoS Med* 13**,** e1002152.

Hussain, R., Dawood, G., Abrar, N., Toossi, Z., Minai, A., Dojki, M., and Ellner, J.J. (1995). Selective increases in antibody isotypes and immunoglobulin G subclass responses to secreted antigens in tuberculosis patients and healthy household contacts of the patients. *Clin Diagn Lab Immunol* 2**,** 726-732.

Hussain, R., Shiratsuchi, H., Ellner, J.J., and Wallis, R.S. (2000). PPD-specific IgG1 antibody subclass upregulate tumour necrosis factor expression in PPD-stimulated monocytes: possible link with disease pathogenesis in tuberculosis. *Clin Exp Immunol* 119**,** 449-455.

Johansson, S.E., Rollman, E., Chung, A.W., Center, R.J., Hejdeman, B., Stratov, I., Hinkula, J., Wahren, B., Karre, K., Kent, S.J., and Berg, L. (2011). NK cell function and antibodies mediating ADCC in HIV-1-infected viremic and controller patients. *Viral Immunol* 24**,** 359-368.

Kalia, K., Sharma, S., and Mistry, K. (2004). Non-enzymatic glycosylation of immunoglobulins in diabetic nephropathy. *Clin Chim Acta* 347**,** 169-176.

Kimuda, S.G., Biraro, I.A., Bagaya, B.S., Raynes, J.G., and Cose, S. (2018). Characterising antibody avidity in individuals of varied Mycobacterium tuberculosis infection status using surface plasmon resonance. *PLoS One* 13**,** e0205102.

Kumagai, T., Palacios, A., Casadevall, A., Garcia, M.J., Toro, C., Tiemeyer, M., and Prados-Rosales, R. (2019). Serum IgM Glycosylation Associated with Tuberculosis Infection in Mice. *mSphere* 4.

Lardinois, O.M., Deterding, L.J., Hess, J.J., Poulton, C.J., Henderson, C.D., Jennette, J.C., Nachman, P.H., and Falk, R.J. (2019). Immunoglobulins G from patients with ANCA-associated vasculitis are atypically glycosylated in both the Fc and Fab regions and the relation to disease activity. *PLoS One* 14**,** e0213215.

Lemmers, R.F.H., Vilaj, M., Urda, D., Agakov, F., Simurina, M., Klaric, L., Rudan, I., Campbell, H., Hayward, C., Wilson, J.F., Lieverse, A.G., Gornik, O., Sijbrands, E.J.G., Lauc, G., and Van Hoek, M. (2017). IgG glycan patterns are associated with type 2 diabetes in independent European populations. *Biochim Biophys Acta Gen Subj* 1861**,** 2240-2249.

Lenzini, L., Rottoli, P., and Rottoli, L. (1977). The spectrum of human tuberculosis. *Clin Exp Immunol* 27**,** 230-237.

Lopez-Parra, V., Mallavia, B., Lopez-Franco, O., Ortiz-Munoz, G., Oguiza, A., Recio, C., Blanco, J., Nimmerjahn, F., Egido, J., and Gomez-Guerrero, C. (2012). Fcgamma receptor deficiency attenuates diabetic nephropathy. *J Am Soc Nephrol* 23**,** 1518-1527.

Lu, L.L., Chung, A.W., Rosebrock, T.R., Ghebremichael, M., Yu, W.H., Grace, P.S., Schoen, M.K., Tafesse, F., Martin, C., Leung, V., Mahan, A.E., Sips, M., Kumar, M.P., Tedesco, J., Robinson, H., Tkachenko, E., Draghi, M., Freedberg, K.J., Streeck, H., Suscovich, T.J., Lauffenburger, D.A., Restrepo, B.I., Day, C., Fortune, S.M., and Alter, G. (2016). A Functional Role for Antibodies in Tuberculosis. *Cell* 167**,** 433-443 e414.

Lubbers, R., Sutherland, J.S., Goletti, D., De Paus, R.A., Van Moorsel, C.H.M., Veltkamp, M., Vestjens, S.M.T., Bos, W.J.W., Petrone, L., Del Nonno, F., Bajema, I.M., Dijkman, K., Verreck, F.a.W., Walzl, G., Gelderman, K.A., Groeneveld, G.H., Geluk, A., Ottenhoff, T.H.M., Joosten, S.A., and Trouw, L.A. (2018). Complement Component C1q as Serum Biomarker to Detect Active Tuberculosis. *Front Immunol* 9**,** 2427.

Luyckx, V.A., Tonelli, M., and Stanifer, J.W. (2018). The global burden of kidney disease and the sustainable development goals. *Bull World Health Organ* 96**,** 414-422D.

Machado, L.R., Bowdrey, J., Ngaimisi, E., Habtewold, A., Minzi, O., Makonnen, E., Yimer, G., Amogne, W., Mugusi, S., Janabi, M., Aderaye, G., Mugusi, F., Viskaduraki, M., Aklillu, E., and Hollox, E.J. (2013). Copy number variation of Fc gamma receptor genes in HIV-infected and HIV-tuberculosis co-infected individuals in sub-Saharan Africa. *PLoS One* 8**,** e78165.

Maglione, P.J., Xu, J., Casadevall, A., and Chan, J. (2008). Fc gamma receptors regulate immune activation and susceptibility during Mycobacterium tuberculosis infection. *J Immunol* 180**,** 3329-3338.

Mallat, Z. (2011). The B-side story in insulin resistance. *Nat Med* 17**,** 539-540.

Mattos, A.M., Chaves, A.S., Franken, K.L., Figueiredo, B.B., Ferreira, A.P., Ottenhoff, T.H., and Teixeira, H.C. (2016). Detection of IgG1 antibodies against Mycobacterium tuberculosis DosR and Rpf antigens in tuberculosis patients before and after chemotherapy. *Tuberculosis (Edinb)* 96**,** 65-70.

Mistry, K., and Kalia, K. (2009). Non enzymatic glycosylation of IgG and their urinary excretion in patients with diabetic nephropathy. *Indian J Clin Biochem* 24**,** 159-165.

Moore, J.S., Wu, X., Kulhavy, R., Tomana, M., Novak, J., Moldoveanu, Z., Brown, R., Goepfert, P.A., and Mestecky, J. (2005). Increased levels of galactose-deficient IgG in sera of HIV-1-infected individuals. *AIDS* 19**,** 381-389.

Nicholas, D.A., Salto, L.M., Boston, A.M., Kim, N.S., Larios, M., Beeson, W.L., Firek, A.F., Casiano, C.A., Langridge, W.H., Cordero-Macintyre, Z., and De Leon, M. (2015). Identification of Anti-Long Chain Saturated Fatty Acid IgG Antibodies in Serum of Patients with Type 2 Diabetes. *Mediators Inflamm* 2015**,** 196297.

Raja, A., Baughman, R.P., and Daniel, T.M. (1988). The detection by immunoassay of antibody to mycobacterial antigens and mycobacterial antigens in bronchoalveolar lavage fluid from patients with tuberculosis and control subjects. *Chest* 94**,** 133-137.

Rodriguez, A., Tjarnlund, A., Ivanji, J., Singh, M., Garcia, I., Williams, A., Marsh, P.D., Troye-Blomberg, M., and Fernandez, C. (2005). Role of IgA in the defense against respiratory infections IgA deficient mice exhibited increased susceptibility to intranasal infection with Mycobacterium bovis BCG. *Vaccine* 23**,** 2565-2572.

Romanowski, K., Clark, E.G., Levin, A., Cook, V.J., and Johnston, J.C. (2016). Tuberculosis and chronic kidney disease: an emerging global syndemic. *Kidney Int* 90**,** 34-40.

Roy Chowdhury, R., Vallania, F., Yang, Q., Lopez Angel, C.J., Darboe, F., Penn-Nicholson, A., Rozot, V., Nemes, E., Malherbe, S.T., Ronacher, K., Walzl, G., Hanekom, W., Davis, M.M., Winter, J., Chen, X., Scriba, T.J., Khatri, P., and Chien, Y.H. (2018). A multi-cohort study of the immune factors associated with M. tuberculosis infection outcomes. *Nature* 560**,** 644-648.

Sadanand, S., Das, J., Chung, A.W., Schoen, M.K., Lane, S., Suscovich, T.J., Streeck, H., Smith, D.M., Little, S.J., Lauffenburger, D.A., Richman, D.D., and Alter, G. (2018). Temporal variation in HIV-specific IgG subclass antibodies during acute infection differentiates spontaneous controllers from chronic progressors. *AIDS* 32**,** 443-450.

Sartain, M.J., Slayden, R.A., Singh, K.K., Laal, S., and Belisle, J.T. (2006). Disease state differentiation and identification of tuberculosis biomarkers via native antigen array profiling. *Mol Cell Proteomics* 5**,** 2102-2113.

Sousa, A.O., Henry, S., Maroja, F.M., Lee, F.K., Brum, L., Singh, M., Lagrange, P.H., and Aucouturier, P. (1998). IgG subclass distribution of antibody responses to protein and polysaccharide mycobacterial antigens in leprosy and tuberculosis patients. *Clin Exp Immunol* 111**,** 48-55.

Tanigaki, K., Sacharidou, A., Peng, J., Chambliss, K.L., Yuhanna, I.S., Ghosh, D., Ahmed, M., Szalai, A.J., Vongpatanasin, W., Mattrey, R.F., Chen, Q., Azadi, P., Lingvay, I., Botto, M., Holland, W.L., Kohler, J.J., Sirsi, S.R., Hoyt, K., Shaul, P.W., and Mineo, C. (2018). Hyposialylated IgG activates endothelial IgG receptor FcgammaRIIB to promote obesity-induced insulin resistance. *J Clin Invest* 128**,** 309-322.

Tecklenborg, J., Clayton, D., Siebert, S., and Coley, S.M. (2018). The role of the immune system in kidney disease. *Clin Exp Immunol* 192**,** 142-150.

Tomaras, G.D., Ferrari, G., Shen, X., Alam, S.M., Liao, H.X., Pollara, J., Bonsignori, M., Moody, M.A., Fong, Y., Chen, X., Poling, B., Nicholson, C.O., Zhang, R., Lu, X., Parks, R., Kaewkungwal, J., Nitayaphan, S., Pitisuttithum, P., Rerks-Ngarm, S., Gilbert, P.B., Kim, J.H., Michael, N.L., Montefiori, D.C., and Haynes, B.F. (2013). Vaccine-induced plasma IgA specific for the C1 region of the HIV-1 envelope blocks binding and effector function of IgG. *Proc Natl Acad Sci U S A* 110**,** 9019-9024.

Tomaras, G.D., and Haynes, B.F. (2009). HIV-1-specific antibody responses during acute and chronic HIV-1 infection. *Curr Opin HIV AIDS* 4**,** 373-379.

Unaids (2019). *Global HIV & AIDS statistics — 2019 fact sheet* [Online]. Available: <https://www.unaids.org/en/resources/fact-sheet> [Accessed].

Vadrevu, S.K., Trbojevic-Akmacic, I., Kossenkov, A.V., Colomb, F., Giron, L.B., Anzurez, A., Lynn, K., Mounzer, K., Landay, A.L., Kaplan, R.C., Papasavvas, E., Montaner, L.J., Lauc, G., and Abdel-Mohsen, M. (2018). Frontline Science: Plasma and immunoglobulin G galactosylation associate with HIV persistence during antiretroviral therapy. *J Leukoc Biol* 104**,** 461-471.

Viana, L.A., Cristelli, M.P., Santos, D.W., Tavares, M.G., Dantas, M.T.C., Felipe, C.R., Silva, H.T., and Pestana, J.M. (2019). Influence of epidemiology, immunosuppressive regimens, clinical presentation, and treatment on kidney transplant outcomes of patients diagnosed with tuberculosis: A retrospective cohort analysis. *Am J Transplant* 19**,** 1421-1431.

Wang, Y., and Tao, Y. (2018). Tuberculosis-associated IgA nephropathy. *J Int Med Res* 46**,** 2549-2557.

Winer, D.A., Winer, S., Shen, L., Wadia, P.P., Yantha, J., Paltser, G., Tsui, H., Wu, P., Davidson, M.G., Alonso, M.N., Leong, H.X., Glassford, A., Caimol, M., Kenkel, J.A., Tedder, T.F., Mclaughlin, T., Miklos, D.B., Dosch, H.M., and Engleman, E.G. (2011). B cells promote insulin resistance through modulation of T cells and production of pathogenic IgG antibodies. *Nat Med* 17**,** 610-617.

World Health Organisation (2018). *TB causes 1 in 3 HIV deaths* [Online]. World Health Organisation. Available: <https://www.who.int/hiv/mediacentre/news/hiv-tb-patient-centred-care/en/> [Accessed].

World Health Organization (2016). "Global Report on Diabetes". (France: World Health Organization).

World Health Organization (2018). "Global Tuberculosis Report 2018". (Geneva: World Health Organization).

Wren, L.H., Chung, A.W., Isitman, G., Kelleher, A.D., Parsons, M.S., Amin, J., Cooper, D.A., Investigators, A.S.C., Stratov, I., Navis, M., and Kent, S.J. (2013). Specific antibody-dependent cellular cytotoxicity responses associated with slow progression of HIV infection. *Immunology* 138**,** 116-123.

Yates, N.L., Liao, H.X., Fong, Y., Decamp, A., Vandergrift, N.A., Williams, W.T., Alam, S.M., Ferrari, G., Yang, Z.Y., Seaton, K.E., Berman, P.W., Alpert, M.D., Evans, D.T., O'connell, R.J., Francis, D., Sinangil, F., Lee, C., Nitayaphan, S., Rerks-Ngarm, S., Kaewkungwal, J., Pitisuttithum, P., Tartaglia, J., Pinter, A., Zolla-Pazner, S., Gilbert, P.B., Nabel, G.J., Michael, N.L., Kim, J.H., Montefiori, D.C., Haynes, B.F., and Tomaras, G.D. (2014). Vaccine-induced Env V1-V2 IgG3 correlates with lower HIV-1 infection risk and declines soon after vaccination. *Sci Transl Med* 6**,** 228ra239.

Yates, N.L., Lucas, J.T., Nolen, T.L., Vandergrift, N.A., Soderberg, K.A., Seaton, K.E., Denny, T.N., Haynes, B.F., Cohen, M.S., and Tomaras, G.D. (2011). Multiple HIV-1-specific IgG3 responses decline during acute HIV-1: implications for detection of incident HIV infection. *AIDS* 25**,** 2089-2097.

Yu, X., Prados-Rosales, R., Jenny-Avital, E.R., Sosa, K., Casadevall, A., and Achkar, J.M. (2012). Comparative evaluation of profiles of antibodies to mycobacterial capsular polysaccharides in tuberculosis patients and controls stratified by HIV status. *Clin Vaccine Immunol* 19**,** 198-208.

Zelmer, A., Stockdale, L., Prabowo, S.A., Cia, F., Spink, N., Gibb, M., Eddaoudi, A., and Fletcher, H.A. (2018). High monocyte to lymphocyte ratio is associated with impaired protection after subcutaneous administration of BCG in a mouse model of tuberculosis. *F1000Res* 7**,** 296.

Zhai, X., Qian, G., Wang, Y., Chen, X., Lu, J., Zhang, Y., Huang, Q., and Wang, Q. (2016). Elevated B Cell Activation is Associated with Type 2 Diabetes Development in Obese Subjects. *Cell Physiol Biochem* 38**,** 1257-1266.

Zimmermann, N., Thormann, V., Hu, B., Kohler, A.B., Imai-Matsushima, A., Locht, C., Arnett, E., Schlesinger, L.S., Zoller, T., Schurmann, M., Kaufmann, S.H., and Wardemann, H. (2016). Human isotype-dependent inhibitory antibody responses against Mycobacterium tuberculosis. *EMBO Mol Med* 8**,** 1325-1339.

Zolla-Pazner, S., Decamp, A., Gilbert, P.B., Williams, C., Yates, N.L., Williams, W.T., Howington, R., Fong, Y., Morris, D.E., Soderberg, K.A., Irene, C., Reichman, C., Pinter, A., Parks, R., Pitisuttithum, P., Kaewkungwal, J., Rerks-Ngarm, S., Nitayaphan, S., Andrews, C., O'connell, R.J., Yang, Z.Y., Nabel, G.J., Kim, J.H., Michael, N.L., Montefiori, D.C., Liao, H.X., Haynes, B.F., and Tomaras, G.D. (2014). Vaccine-induced IgG antibodies to V1V2 regions of multiple HIV-1 subtypes correlate with decreased risk of HIV-1 infection. *PLoS One* 9**,** e87572.
